# A highly infective plant-associated bacterium influences reproductive rates in pea aphids

**DOI:** 10.1098/rsos.150478

**Published:** 2016-02-10

**Authors:** Tory A. Hendry, Kelley J. Clark, David A. Baltrus

**Affiliations:** School of Plant Sciences, University of Arizona, Tucson, AZ 85721, USA

**Keywords:** *Acyrthosiphon pisum*, *Pseudomonas syringae*, fecundity compensation

## Abstract

Pea aphids, *Acyrthosiphon pisum*, have the potential to increase reproduction as a defence against pathogens, though how frequently this occurs or how infection with live pathogens influences this response is not well understood. Here we determine the minimum infective dose of an environmentally common bacterium and possible aphid pathogen, *Pseudomonas syringae*, to determine the likelihood of pathogenic effects to pea aphids. Additionally, we used *P. syringae* infection to investigate how live pathogens may alter reproductive rates. We found that oral bacterial exposure decreased subsequent survival of aphids in a dose-dependent manner and we estimate that ingestion of less than 10 bacterial cells is sufficient to increase aphid mortality. Pathogen dose was positively related to aphid reproduction. Aphids exposed to low bacterial doses showed decreased, although statistically indistinguishable, fecundity compared to controls. Aphids exposed to high doses reproduced significantly more than low dose treatments and also more, but not significantly so, than controls. These results are consistent with previous studies suggesting that pea aphids may use fecundity compensation as a response to pathogens. Consequently, even low levels of exposure to a common plant-associated bacterium may therefore have significant effects on pea aphid survival and reproduction.

## 1. Introduction

The plant-associated bacterium *Pseudomonas syringae* (Gammaproteobacteria) is a common plant pathogen and is also frequently found epiphytically on leaf surfaces without causing disease symptoms [1,2]. Recent studies have found that *P. syringae* can also be a highly virulent pathogen of two hemipteran insects, pea aphids (*Acyrthosiphon pisum*, Hemiptera: Aphididae) and sweet potato whiteflies (*Bemisia tabaci*, Hemiptera: Aleyrodidae) [3,4]. In both species, oral exposure to two divergent *P. syringae* strains, suspended in artificial diet, leads to bacterial growth in the insect and up to 100% mortality within a few days. Although these studies showed that insects could become infected while feeding on plants with epiphytic *P. syringae* populations, how commonly insects become infected in nature is unknown. Previous work has relied on high bacterial doses and artificial diet, making it unclear if the minimum infective dose of *P. syringae* in insects is within a naturally occurring range for bacterial population sizes, or if insects feeding on healthy plants are better able to defend against, or compensate for, the pathogen. For instance, feeding behaviour or limitations in the size of the digestive tract may constrain the number of bacteria that can be ingested by many hemipteran insects [5,6] and bacterial population sizes may fluctuate dramatically in nature [1], making it unclear how frequently insects are infected by pathogenic strains of plant-associated bacteria.

A number of virulent fungal pathogens of aphids are known but comparatively few bacterial pathogens have been described [5]. This may result in part from observation biases, as bacterial pathogens may be asymptomatic or cause sporadic infections [3]. Hemipteran insects regularly encounter epiphytic bacteria on plant surfaces as they feed and these bacteria can be isolated from surface sterilized insects [7–9]. Several of these plant-associated bacteria have been found to also be highly virulent to pea aphids [10–12], suggesting that in fact the phyllosphere may be an important reservoir for virulent bacterial pathogens of hemipteran insects.

A high prevalence of virulent pathogens that are difficult to defend against immunologically could lead to the evolution of non-immunological defences in hosts [13]. Pea aphids may use multiple non-immunological defences against pathogens, including symbiont-mediated defence and fecundity compensation [14]. For instance, hemipteran insects can benefit from a number of secondary endosymbionts that may defend against pathogens [3,15,16]. Additionally, fecundity compensation, increasing reproductive rate in response to infection, has been described in a number of organisms and is thought to be adaptive when an immunological defence is unlikely to lead to survival [13,17–21]. Fecundity compensation may be particularly useful for aphids owing to their high rates of asexual reproduction [22] and reduced number and expression of immune genes [23–25]. Although the potential for this response has been found in pea aphids after exposure by stabbing with heat killed pathogens, it has not been explored using live pathogens, so it remains unclear how common or adaptive the response would be in nature [13,22,26].

In this study we use *P. syringae* as an example of a common pathogen with the potential to be highly virulent to pea aphids. In order to assess the potential for *P. syringae* to infect and kill insects we use pathogen exposure via artificial diet to control pathogen dose and determine if the minimum infective dose of *P. syringae* in pea aphids is within naturally occurring bacterial population size ranges. We also investigated how pea aphids respond to infection, particularly with regards to fecundity responses, by orally exposing aphids to varying doses of *P. syringae* and then tracking their survival, development time, and reproduction rates on healthy plants.

## 2. Material and methods

### 2.1 Bacterial strains and aphid rearing

Two *P. syringae* strains previously shown to be virulent to pea aphids, *P. syringae* pv. *syringae*B728a (*Psy* B728a) and pv. *tomato* DC3000 (*Pto* DC3000), were used in this study. These strains were obtained from Steven Lindow (*Psy* B728a) and Allan Collmer (*Pto* DC3000) and have been previously selected for rifampicin resistance. Clonal pea aphid colonies (*A. pisum*clone 5A, collected by N. Moran in 1999 in Madison, WI) were kept on fava bean plants at 24°C with 16 h of light and 8 h of darkness. This clone harbours the obligate symbiont *Buchnera*, but no secondary symbionts that might defend against pathogens [27]. To limit maternal effects or health differences between plants, 5–10 adults from different plants were distributed among 10 two-week-old plants, and allowed to multiply to high density for 5–7 days. For experiments, second and third instar aphids were collected from healthy plants and divided into treatments so that each treatment received approximately the same number of individuals from each of the collection plants.

### 2.2 Determining the minimum infective dose of *Pseudomonas syringae*

We sought to determine the minimum infective dose of bacterial strain *Psy* B728a, as it is a common epiphyte likely to be encountered by insects on leaves [1,28], and is also highly virulent to aphids, making mortality differences between doses more apparent [3,4]. To control bacterial dosage, we used *in vitro* pathogenicity assays as described previously [3]. Wells of a 96-well plate were filled with 200 μl of artificial aphid diet [29] and the plate was covered with parafilm to make a feeding sachet. Artificial diet was either mixed with 10 mM MgCl_2_ as a negative control or 10 mM MgCl_2_ solutions containing varying cell titers of bacteria. To make bacterial treatments, overnight cultures of bacteria were pelleted, washed once with 10 mM MgCl_2_ and resuspended in the same solution. Optical density measurements were used to approximate cell density and then the resuspended culture was serially diluted to 0, 10^−2^, 10^−4^, 10^−6^, or 10^−8^ of the original starter culture. These cell suspensions were mixed with artificial diet in a ratio of 1 ml cell suspension in 5 ml of diet and checked to determine cell titer of the final solution by serial dilution and colony counts. Previous work found that the *P. syringae* strains used here do not grow well in artificial aphid diet and therefore bacterial titers remain fairly constant over a 24-hour exposure period ([3]; T. A. Hendry 2012, unpublished data). Treatments with each bacterial titer were replicated three times, with the exception of the 1/10^8^ dilution, which was replicated twice, over the course of six experiments. Five out of six experiments included a negative control treatment with no bacteria and each experiment included a range of doses from low to high. This range was lowered over the course of experiments until doses that did not impact aphid survival were reached.

For each replicate treatment 30–50 second and third instar aphids were placed individually in wells of a 96-well plate and the feeding sachet plate was inverted above them, allowing the insects to feed through the parafilm and keeping them restricted to individual wells. Experimental aphids were kept under the same environmental conditions described for aphid colonies. After the aphids fed for 24 h the feeding sachet was replaced with a new one containing sterile artificial diet and a new sterile sachet was provided every 24 h for four days. At the time that the sachet was replaced, aphids were also checked for mortality. An aphid was counted as dead if it had turned brown or was at the bottom of the well and did not move during the observation. If an aphid was on the parafilm of the feeding sachet but not moving it was assumed to be feeding and alive. We calculated the number of bacterial cells likely to have been consumed by aphids using colony count estimates of the bacterial titer present in the artificial diet and published estimates of the volume of diet likely to have been consumed by the aphids. Using the estimates of Auclair [30] we calculated low and high (0.69 to 2.5 μl) estimates of diet consumption within a 24 h period based on the lowest and highest observed volumes of honeydew secreted by third instar to adult pea aphids feeding on various varieties of pea plants. While these estimates are no doubt different than the actual volume of artificial diet consumed in our study, we feel that it is unlikely that aphids consumed an amount exceeding these estimates that would alter our conclusions.

We performed two checks to confirm that aphid death was related to bacterial exposure. First, we sampled aphids in each bacterial treatment to determine the percentage of individuals that were infected. For each treatment replicate, eight aphids that died between 48 and 72 h after the start of the experiment were homongenized individually in 100 μl volumes of 10 mM MgCl_2_. The resulting mixtures were plated on King's B agar plates containing rifampicin and all samples scored positive for infection grew a lawn of colonies in 10 μl of the sample. Additionally, to confirm that aphid death was not greatly increased by the *in vitro* conditions, we performed one semi-*in vivo* experiment where aphids were exposed to undiluted *Psy* B728a, a 10^−2^ dilution, a 10^−4^ dilution, or a control, in artificial diet using the procedures described above. After 24 h on artificial diet aphids were transferred to healthy two-week old fava bean plants, one plant and 30–40 aphids per treatment, and kept under the conditions described above. The number of surviving aphids on each plant was determined after three days on plants.

### 2.3 Infected aphid survival, development and reproduction on plants

We sought to determine effects of *P. syringae* on pea aphid survival, development time and reproduction under more natural conditions by following infected aphids transferred to healthy plants. In order to track dose-dependent effects over a longer timescale, aphids in these experiments were exposed to varying doses of a low virulence strain, *Pto* DC3000, in artificial diet as described above. Four replicate experiments were performed independently, with each including a control treatment of aphids exposed to no bacteria, as well as 3–4 treatments in which aphids were exposed to bacteria at concentrations ranging from 1–10^8^ colony forming units (CFU) ml^−1^, with bacterial concentrations controlled as described above. After 24 h of oral exposure third instar aphids from each treatment were gently placed on healthy 2-week-old fava bean plants with 35–40 aphids per plant. This procedure was replicated four times with each experiment consisting of a negative control treatment and dilutions. The total number of surviving aphids, the number of adults, and the number of newly born aphids on plants were counted daily for seven days. These values were used to calculate the mean development time of aphids from placement on plants to adulthood. Newly born offspring were counted daily and allowed to remain on the plants. The daily per capita asexual reproductive output was calculated using daily births and the number of adults still alive on that day. The daily reproductive output per living adult aphid was summed across all days to produce a total for the experiment. We note that because aphids were placed in groups on plants all values are means for the aphids in a given treatment rather than individual values.

### 2.4 Statistical analysis

In each survival experiment, survival was modelled in R [31] using Cox proportional hazards survival analysis. Differences in survival between treatments were tested for significance using log-rank tests. The effect of pseudoreplicate (different bacterial doses or control treatments across experiments performed at different times) was included in the analysis as a random variable. Treatments were generally consistent across experiments (electronic supplementary material, table S1).

Basic linear regression was used to investigate the relationships between dose and infection, infection and survival, dose and development time, as well as survival and reproduction. Bacterial dose was log_10_ transformed and proportions were arcsine transformed. Linear regression was also used to investigate the relationship between bacterial dose and total asexual reproduction per capita, but very high doses that lead to almost no reproduction were excluded from analysis. To further test the effect of different doses on reproduction, doses that lead to statistically indistinguishable aphid survival at the end of the experiment, based on survival curves, were grouped together as low doses (10–10^3^ CFU ml^−1^), medium doses (10^4^–10^5^ CFU ml^−1^), high doses (10^7^ CFU ml^−1^), and very high doses (10^8^ CFU ml^−1^, a dose whose survival was not significantly different from high doses, but killed nearly all aphids before reproduction). These were then analysed using ANOVA to determine effects of dose on reproduction. In order to determine if different doses influenced the timing of reproduction, we investigated the proportion of reproduction that took place after six versus seven days on plants. The majority of reproduction took place on the sixth and seventh days and treatments showed no obvious differences in reproduction before day six. The proportion of reproduction on day six versus on day seven was analysed using a z-ratio test for difference between proportions.

## 3. Results

### 3.1 Dose dependent effects of *Pseudomonas syringae* on pea aphids

**Figure 1.**
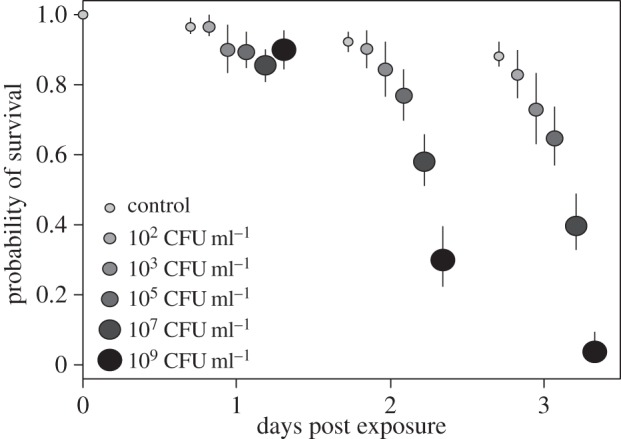
Probability of survival of aphids exposed to varying doses of *Psy* B728a. The center of dots represents the probability of survival as calculated using Cox proportional hazard analysis and lines show the 95% confidence interval around that estimate. Data are shown for days 1, 2, and 3, with points staggered for clarity.

Increasing doses of *Psy* B728a led to increased probability of mortality in pea aphids (table 1, figure 1 and electronic supplementary material S2). This pattern became more pronounced over time with the largest differences between doses appearing on day three. Four days after the start of the experiment similar patterns between doses were also found, though the pattern was less pronounced owing to high overall mortality (data not shown). The highest bacterial doses, 10^5^, 10^7^ and 10^9^ CFU ml^−1^, resulted in significantly different survival compared to other doses by day three (table 1). The lower doses, 10^2^ and 10^3^ CFU ml^−1^, resulted in survival probabilities that were lower than control treatments but not significantly different from each other (table 1). The fact that these experiments were done under laboratory conditions with artificial diet did not seem to greatly impact the results, as aphids that were infected with various doses of *Psy* B728a and then placed on healthy plants showed similarly scaled dose dependent decreases in survival and generally survival did not differ between artificial versus on plant experiments with the same dose (electronic supplementary material, table S3). We note, however, that aphid feeding may have a negative impact on plants, which may indirectly effect aphid health and counteract any benefit they receive from feeding on plants versus diet. However,since the plants appeared healthy and were not overwhelmed with aphid numbers, this effect may be minimal in the timeframe of these experiments.

**Table 1.** Survival and cells probably ingested for each dose of *Psy* B728a. (Statistics are based on likelihood ratio tests and Cox proportional hazard survival analysis.)

treatment	d.f.	*χ*^2^	survival range^a^	cells ingested^b^
control			0.83–0.91	
10^2^ CFU ml^−1^	1	26.6^d^	0.63–0.84	0.05–0.2
10^3^ CFU ml^−1^	1	50.7^d^	0.57–0.74	2.5–9.2
10^5^ CFU ml^−1^	1	85.6^d^	0.77–0.90^c^	211–770.8
10^7^ CFU ml^−1^	1	101.1^d^	0.32–0.49^c^	27 273–99 651.2
10^9^ CFU ml^−1^	1	130.0^d^	0.06–0.14^c^	n.a.

^*a*^95% confidence intervals for survival probability at 72 h post exposure.

^*b*^Based on estimates of volume of diet ingested [30].

^c^Significantly different survival compared to other doses.

^d^Decreased survival compared to control; *p*<0.05.

### 3.2 Minimum infective dose of *Psy* B728a to pea aphids

**Figure 2.**
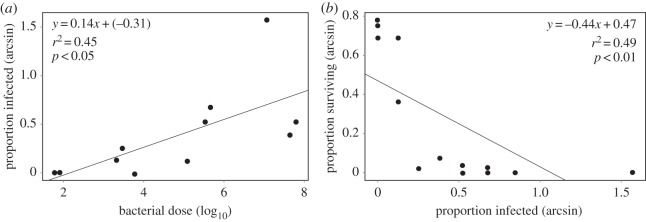
Relationships between bacterial dose and incidence of infection (*a*) as well as the relationship between infection incidence and survival (*b*). Points represent the values observed in each independent treatment and the proportion of individuals surviving at 96 h post exposure was used for survival.

Increasing doses of *P. syringae* corresponded to an increased likelihood of infection (figure 2*a*; *r*^2^=0.45, *F*_1,9_=7.22, *p*<0.05) and increasing infection likelihoods were related to decreased survival (figure 2*b*; *r*^2^=0.49, *F*_1,12_=11.55, *p*<0.01), suggesting that infection and growth of bacteria inside insects lead to mortality, as seen in previous work [3,4]. Together these results suggest that large doses increased the chances of infection and thus decreased survival, rather than the size of the dose directly influencing survival. To determine the minimum infective dose of *Psy* B728a needed to cause mortality in pea aphids we used previously estimated volumes of food consumed by the aphids to calculate the number of cells that were probably ingested by aphids in each treatment (table 1). The lowest two doses both significantly increased mortality compared to control treatments, but were not significantly different from each other (table 1). We therefore combined these doses to determine that a range of 1–9 cells ingested was probably sufficient to increase mortality. This suggests that the minimum infective dose of *P. syringae* to pea aphids may be very low.

### 3.3 Survival and growth of *Pto* DC3000 infected aphids on plants

**Figure 3.**
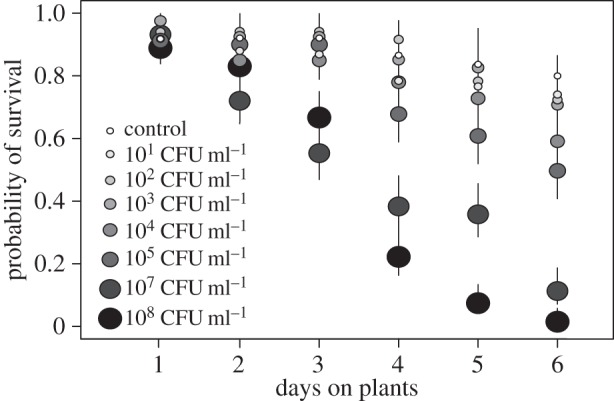
Probability of survival of aphids exposed to varying doses of *Pto* DC3000. The centre of dots represent the probability of survival as calculated using Cox proportional hazard analysis and lines show the 95% confidence interval around that estimate.

The less virulent strain *Pto* DC3000 also appears to cause dose dependent mortality, based on the pattern of survival for different treatments over time (figure 3). In these experiments, where aphids were fed on healthy plants, mortality was lower and occurred over a longer timescale than after *Psy* B728a exposure, as has been shown previously [3,4]. Infection rates were not tested in this experiment, but dose is presumed to also correspond to increased infection rates with this strain. Dose was not significantly related to the time aphids took to develop to adulthood after placement on plants (electronic supplementary material, figure S1; *r*^2^=0.09, *F*_1,21_=2.16, *p*=0.16), from which we infer infection does not effect development time if infection occurs in second or third instar aphids.

### 3.4 Dose dependent effects on pea aphid reproduction

**Figure 4.**
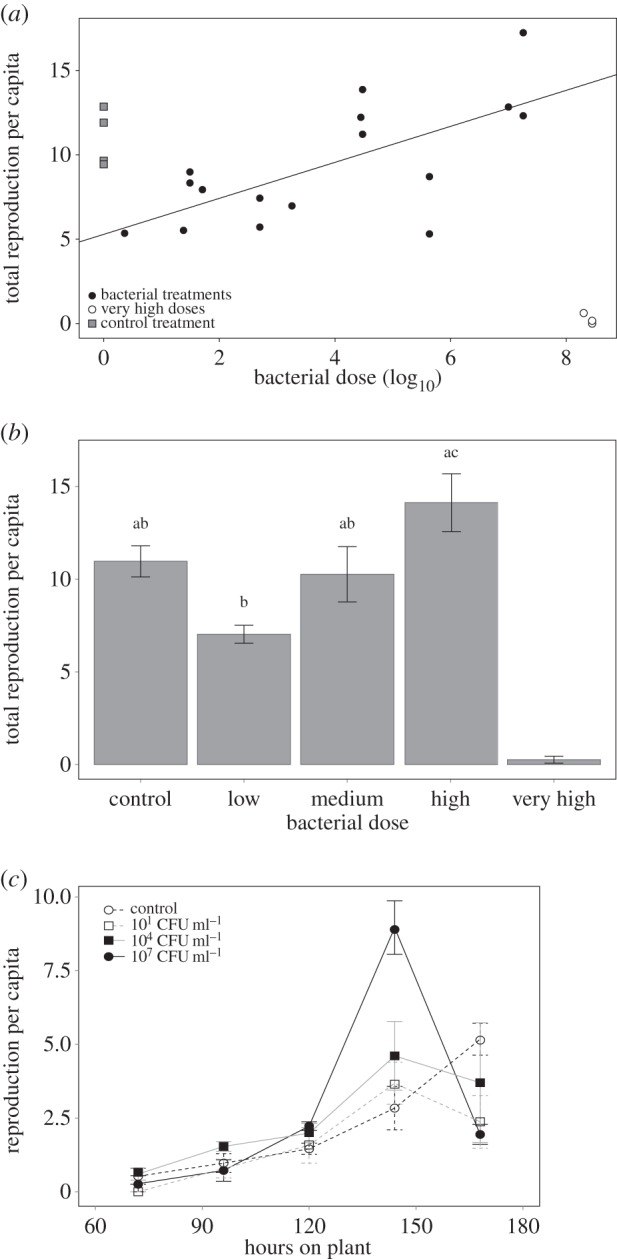
The relationship between total asexual reproduction per capita and bacterial *Pto* DC3000 dose in each treatment (*a*) as well as asexual reproduction across categories of doses (low, medium, high, very high) (*b*), and reproduction per day averaged across similar (CFU ml^−1^ within a factor of 10 from each other) doses (*c*). In (*a*) a regression line (*y*=1.07*x*+5.29, *r*^2^=0.47, *p*<0.01) was fitted to show the relationship between dose and reproduction for all bacterial dose treatments excluding very high treatments with very little reproduction. In (*b*) error bars show standard error and letters indicate significant differences in ANOVA. In (*c*) points show the average number of nymphs born since the last count and error bars show standard error for three dose treatments in each category.

Bacterial dose was positively related to asexual reproduction per aphid. The highest doses used in this experiment, 10^8^ CFU ml^−1^, caused high mortality (figure 3) and few aphids reproduced before death (figure 4*a*). The remaining bacterial doses, ranging from 10 to 10^7^ CFU ml^−1^, were positively related to reproduction, with aphids receiving lower doses reproducing less than control aphids and aphids receiving high doses reproducing more than control aphids (figure 4*a*; *r*^2^=0.47, *F*_1,14_=12.38, *p*<0.01). When these doses were grouped as ‘low’ (10–10^3^ CFU ml^−1^), ‘medium’ (10^4^–10^5^ CFU ml^−1^), ‘high’ (10^7^ CFU ml^−1^), or ‘very high’ (10^8^ CFU ml^−1^, doses which lead to almost no reproduction), low and high treatments were found to have significantly different reproduction per capita and the very high treatment, which had nearly zero reproduction, was significantly lower compared to all other treatments (figure 4*b*; *F*_4,18_=19.68, *p*<0.001). Although they were different from each other, reproduction in both low and high dose treatments was statistically indistinguishable from control reproduction. We note that this study analysed per capita reproduction of all aphids in a treatment, not individual aphid reproduction, so it is possible that aphids reproduced more in treatments where there was higher mortality, and therefore less aphid density on a plant. However, we feel that density differences are unlikely to fully account for this pattern. Firstly, reproductive rate does not have a negative correlation with density as would be expected if high densities decreased reproduction (electronic supplementary material, figure S3). Secondly, aphid densities became high and varied by dose only in the last 1–3 days of the experiment, making density less likely to impact reproduction than factors present at the start of the experiment such as bacterial dose.

Aphids exposed to bacteria tended to reproduce earlier than control aphids (figure 4*c*). This trend was seen in low, medium and high doses but was particularly strong in the high treatment where aphids had a large peak of reproduction six days after exposure and reproduction dropped on day seven, whereas control aphids did not begin to peak in reproduction until day seven. Aphids exposed to high bacterial doses had a significantly higher proportion of reproduction take place on day six than on day seven, compared to control aphids (*F*_3,16_=10.3, *p*<0.001). As no relationship between dose and time to adulthood was found, this trend may not be due to bacterially exposed aphids reaching reproductive age faster than controls. However, we note that two of the high bacterial treatments had the shortest development times of all treatments, so it is possible that only high doses increase development time and not enough of those were included here to show a pattern (electronic supplementary material, figure S1).

## 4. Discussion

This study provides evidence that one virulent strain of *P. syringae* is highly infective to pea aphids, with a minimum infective dose calculated to be lower than 10 cells. Our results indicate that exposure to higher doses of bacteria increase the likelihood of infection, and that ingestion of even a small number of cells significantly increases the chances of aphid death. This strain of *P. syringae* belongs to a clade of strains that are commonly epiphytic and may be found in populations as high as 10^5^ or 10^6^ CFU leaf^−1^, so it is reasonable that aphids may regularly encounter virulent and infective *P. syringae* strains while feeding [1,32]. A previous study found that pea aphids do become infected, at an incidence of about 15–29%, when feeding on plants with high numbers of epiphytic *P. syringae* [4]. Given that a low number of infecting cells are apparently capable of causing death, it seems possible that aphid populations could suffer from exposure to lower *P. syringae* densities on plants as well. A similarly low infective dose, around 10 cells, was also found for the plant pathogen *Dickeya dadantii* in pea aphids [11], so this may be a trend common to other plant-associated bacteria that cause death in hemipteran insects.

By testing a range of doses of a low virulence *P. syringae* strain we found a significant positive relationship between pathogen dose and asexual reproduction in pea aphids, demonstrating that pathogen infection influenced reproductive rate. With increasing dose, reproduction increased until treatments with high doses had higher reproduction than control treatments. Very high doses of this strain led to high mortality and very little aphid reproduction. Although the reproduction in high dose treatments was not significantly higher than controls, the difference in reproduction between low and high doses was significant, suggesting that different effects may be occurring at low versus high doses. We also saw a decrease in reproduction after exposure to low doses, which, although not significant, is intriguing as it may suggest that some cost of infection impedes reproduction. For instance, although pea aphids appear to lack some known immune pathways and show reduced immune responses compared to other insects [23–25] they can mount an immune response to bacteria [26,33–35] and this could create a trade-off between an immune response to infection and reproduction.

The increase in reproduction with increasing dose is consistent with fecundity compensation, or investment in reproduction rather than immune response to a pathogen. Our results cannot eliminate the possibility that aphid density negatively influences reproduction, as higher dose treatments had lower survival and therefore lower aphid density, which could lead to increased reproduction. However, we do not find that density and reproductive rate are negatively correlated. Owing to reproductive rate increasing with time, reproduction is positively related to density when all days are analysed, and no significant relationship is found when days with the highest reproductive rate in each treatment are included. Although no general trend towards low densities increasing reproduction was found, days from high dose treatments appear to be outliers with low density and high reproduction (electronic supplementary material, figure S3). This suggests that these treatments are different in some way, possibly owing to exposure to high levels of the pathogen. Furthermore, aphid densities only became high and variable in the last two days of the experiment and after reproduction had already begun, so density may be a less likely explanation for the pattern observed than bacterial exposure, which occurred four days before reproduction.

This work complements previous studies which found an increase in pea aphid reproduction owing to wounding mediated introduction of heat killed pathogens, as well as owing to wounding alone [13,22,26]. These responses appear to be context dependent and vary with aphid lineage as well as the type of pathogen [13,22]. To our knowledge, this study is the first demonstration consistent with increased fecundity in pea aphids after infection by a live pathogen, suggesting that the response could be used in nature. Our results indicate that infection by pathogens may impact aphid fecundity in different ways depending on the level of exposure or infection. Our results are consistent with a trade-off between immune response and investment in fecundity, where aphids exposed to low doses reproduced less, perhaps because they were allocating energy towards the immune system, and aphids exposed to high doses invested more in reproduction during the timespan of the experiment. These results match theoretical predictions of how increasing infection rates could lead to increased reproduction, i.e. if infection and death are likely, individuals should invest in reproduction over defence [13].

These data suggest that pathogen infection or exposure may have influenced the timing of reproduction, with aphids exposed to high doses reproducing significantly faster than controls. A similar pattern of faster reproduction after pathogen exposure was found for some aphid clones, including the lineage used here, 5A, in previous work [13]. Earlier reproduction lead to overall larger numbers of offspring for high dose treatments than controls, although we note that aphids exposed to high doses greatly decreased reproduction at the same time that control aphids were increasing reproduction, so it is possible that over a longer timeframe this difference could disappear or control aphids could reproduce more. Aphids exposed to low or medium doses also showed a trend of reproducing slightly faster than controls and also decreased in reproduction as control aphids were increasing reproduction, so they reproduced less than aphids in high dose treatments. However, aphids exposed to lower doses may have a better chance of surviving infection longer and reproducing more over longer timescales. It is therefore unclear how beneficial a fecundity compensation response would be in this scenario.

In this context fecundity compensation also has the potential to be indirectly beneficial to pathogens, by producing more offspring that are available hosts and possibly influencing the dynamics of how fecundity responses affect aphid fitness. Because we did not track individual aphid reproduction, it is not possible to tell from these data if individual aphids became infected and subsequently reproduced more, or if uninfected aphids were able to respond to the presence of infected individuals in the population and then increase reproduction. The fact that there was a decrease in reproduction with low doses suggests that responses may have been at the level of the individual.

With the exception of very arid regions, *P. syringae* is a common pathogen and epiphyte on nearly all plants worldwide [1,36]. It can reach relatively high population sizes on leaf surfaces, making it likely that aphids feeding on plants encounter this bacterium. Given that pea aphids can become infected from feeding on plants [4] and are likely to die after ingestion of only a few cells, *P. syringae*, and possibly other plant associated bacteria, have the potential to significantly impact survival of aphid populations. Furthermore, the abundance of these virulent pathogens may increase selection for a plastic reproductive response and fecundity compensation in aphids, and therefore influence the population dynamics of both the aphids and the pathogen.

## Supplementary Material

Supplemental Data tables S1.

## Supplementary Material

Supplemental Table S2 and Figures S1-S3.

## Data accessibility

Datasets supporting this article have been uploaded as part of the electronic supplementary material, table S1.
