# Supplementary material for: A highly infective plant-associated bacterium influences reproductive rates in pea aphids
Source: R Soc Open Sci. 2016 Feb 10;3(2):150478. doi: 10.1098/rsos.150478 (PMC4785972; doi:10.1098/rsos.150478)
Supplement: Supplemental Table S2 and Figures S1-S3. [file rsos150478supp2.docx]

**Table S2.** Comparison of *Psy* B728a infected aphids fed on healthy plants versus artificial diet.

________________________________________________________________________

plants vs. diet^b^ vs. control

Treatment Survival^a^ d.f. χ^2^ d.f. χ^2^

On plants

Control 0.84 1 1.53

10^9^ CFU/mL 0.06 1 0.19 **1 47.95^*^**

10^7^ CFU/mL 0.08 **1 4.77^*^ 1 49.86^*^**

10^5^ CFU/mL 0.24 1 1.95 **1 30.05^*^**

On diet

Control 0.71

10^9^ CFU/mL 0.04 **1 37.31^*^**

10^7^ CFU/mL 0.26 **1 13.45^*^**

10^5^ CFU/mL 0.38 **1 7.3^*^**

* p < 0.05

a Proportion of surviving aphids four days post exposure, calculated by survival analysis

^b^ Comparison of aphids receiving the same dose then fed on plants versus artificial diet

^c^ Statistics based on likelihood ratio tests and Cox proportional hazard survival analysis

**Figure S1**. The relationship between dose of *Pto* DC3000 and development time. Development time is measured in days from placement on plants to adulthood.


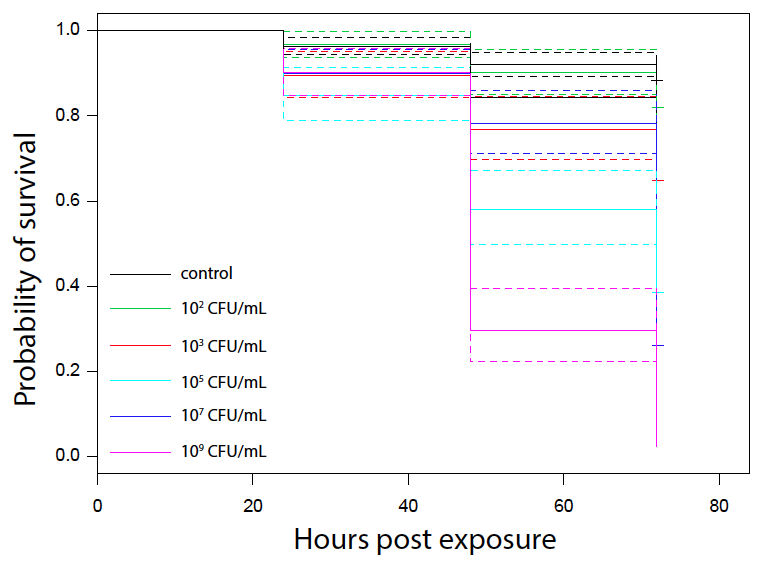


**Figure S2.** Survival curve of aphids exposed to varying doses of *Psy* B728a as calculated using Cox proportional hazard analysis (solid lines). Dotted lines show the 95% confidence interval around the survival estimate.

**Figure S3.** (a) Aphid density versus reproductive rate for all time points. A regression (*y* = 0.013x + 0.515, *r*^2^ = 0.30, *F*_(1,113)_ = 47.81, *p* < 0.001) shows a significant positive relationship between reproduction and density. The total number of aphids per plant, including adults and nymphs, was used.

**Figure S3.** (b) Aphid density versus reproductive rate for days with the highest reproductive rate in each treatment. A regression found no significant relationship between reproduction and density (*r*^2^ = 0.02, *F*_(1,20)_ = 0.35, *p* = 0.56). The total number of aphids per plant, including adults and nymphs, was used.
